# Predictors and the Subsequent Risk of End-Stage Renal Disease – Usefulness of 30% Decline in Estimated GFR over 2 Years

**DOI:** 10.1371/journal.pone.0132927

**Published:** 2015-07-15

**Authors:** Wen Xiu Chang, Shinichiro Asakawa, Daigo Toyoki, Yoshikazu Nemoto, Chikayuki Morimoto, Yoshifuru Tamura, Tatsuru Ota, Shigeru Shibata, Yoshihide Fujigaki, Zhong Yang Shen, Shunya Uchida

**Affiliations:** 1 Department of Internal Medicine, Teikyo University School of Medicine, Tokyo, Japan; 2 Department of Nephrology, Tianjin First Central Hospital, Tianjin, China; 3 Department of Organ Transplantation, Tianjin First Central Hospital, Tianjin, China; University of Florida, UNITED STATES

## Abstract

**Background:**

A goal of searching risk factors for chronic kidney disease (CKD) is to halt progressing to end-stage renal disease (ESRD) by potential intervention. To predict the future ESRD, 30% decline in estimated GFR over 2 years was examined in comparison with other time-dependent predictors.

**Methods:**

CKD patients who had measurement of serum creatinine at baseline and 2 years were enrolled (n = 701) and followed up to 6 years. Time-dependent parameters were calculated as time-averaged values over 2 years by a trapezoidal rule. Risk factors affecting the incidence of ESRD were investigated by the extended Cox proportional hazard model with baseline dataset and 2-year time-averaged dataset. Predictive significance of 30% decline in estimated GFR over 2 years for ESRD was analyzed.

**Results:**

For predicting ESRD, baseline estimated GFR and proteinuria were the most influential risk factors either with the baseline dataset or the 2-year time-averaged dataset. Using the 2-year time-averaged dataset, 30% decline in estimated GFR over 2 years by itself showed the highest HR of 31.6 for ESRD whereas addition of baseline estimated GFR, proteinuria, serum albumin and hemoglobin yielded a better model by a multivariate Cox regression model. This novel surrogate was mostly associated with time-averaged proteinuria over 2 years with the cut-off of ~1 g/g creatinine.

**Conclusion:**

These results suggest that decline in estimated GFR and proteinuria are the risk factors while serum albumin and hemoglobin are the protective factors by the time-to-event analysis. Future incidence of ESRD is best predicted by 30% decline in eGFR over 2 years that can be modified by intervention to proteinuria, hemoglobin, uric acid, phosphorus, blood pressure and use of renin-angiotensin system inhibitors in the follow-up of 2 years.

## Introduction

A final goal of chronic kidney disease (CKD) clinic is to inhibit entering dialysis therapy because one may be still satisfied if patients do not reach end-stage renal disease (ESRD) despite continuously showing renal dysfunction such as CKD stage 5. A line of evidence has unexceptionally showed major risk factors of subsequent incidence of (ESRD) such as anemia, proteinuria, hypertension in addition to precedent kidney dysfunction [1–4]. However, the second line risk predictors remain to be unveiled with candidates being hypoalbuminemia, hyperuricemia, hyperphosphatemia, metabolic acidosis, dyslipidemia and diabetes [1,5–11]. The search of the progressing factors of CKD may help halt progressing to ESRD through intervening treatments. Based on the evidence, most treatment guidelines such as KDIGO recommend multidisciplinary interventions towards the final common pathway directing ESRD except for untreatable demographic factors such as age, sex, body height and original kidney diseases [12,13].

Inconsistent results may be attributed to the study design of the previous reports that mainly used the baseline values. Obviously, regarding time-varying risk factors, the continued exposure to a given risk factor can better be represented by a time-averaged value rather than a baseline value. Time-varying risk factors are conceptually grouped into three patterns. First, a factor that remains as a continuous risk over the clinical course such as hypertension, proteinuria, low albumin and diabetes. Second, a factor that is within normal range in the early stage of CKD but deranges with advancement of CKD stage, then in turn affects on the remaining kidneys as a risk factor such as hemoglobin, uric acid, phosphorus and metabolic acidosis. Lastly, lowered baseline estimated GFR is grouped to the third type in which the renal dysfunction worsens in a vicious cycle. This finding is evidenced by the steeper decline in estimated GFR with development of CKD stage [14].

A goal to treat CKD patients is to stop reaching ESRD. Previous clinical trials traditionally used doubling of serum creatinine, ESRD and/or death as primary end points for the risk analysis of CKD progression [15,16]. Doubling of serum creatinine enhanced executing clinical trials but they still needs certain amount of participants and a long period of follow-up [17]. Given this fact, many investigators or drug companies hesitate to perform large-scales randomized clinical trials partly due to cost effectiveness. Having these as a background, it is recently reported that 30% decline in estimated GFR over 2 years can substitute for doubling of serum creatinine (corresponding to 57% decline in estimated GFR over 2 years) [18]. However, this smaller decline in estimated GFR remains to be validated before being widely accepted.

The aim of the present study was to identify the predictors for subsequent ESRD together with usefulness of 30% decline in estimated GFR over 2 years as a novel surrogate marker using 2-year time-averaged dataset.

## Materials and Methods

### Study protocol and ethical statement

We have constructed a retrospective CKD cohort. Inclusion criteria consisted of CKD stage 3 and 4, age 20 to 84 years and observation period ≥ 2 years. On the other hand, patients with nephrotic syndrome, malignancy, obstructive nephropathy or acute kidney injury at entry were excluded. All the patients were followed as long as 6 years until reaching the initiation of dialysis or being censored. The present study was approved by the institutional review board (IRB) in the Teikyo University Review Board #14–115 and was executed in accordance with the principle of the Helsinki Declaration. Written informed consent was waived after approval of IRB and the patient records and information was anonymized and de-identified prior to analysis.

### Parameters analyzed

**Fig 1:**
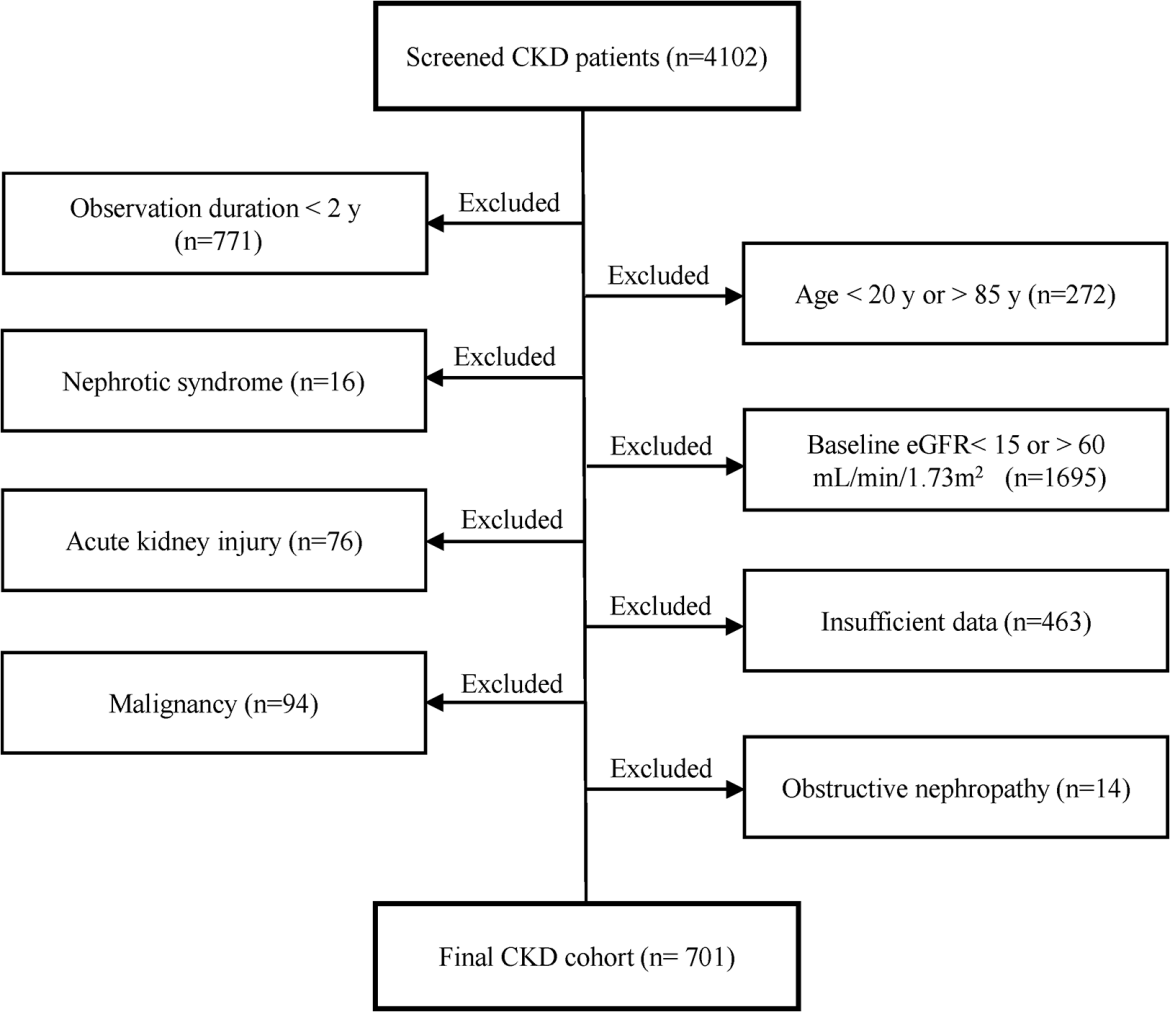
Flow chart of constructing the retrospective CKD cohort.

In the present study we focused on the patient having two measurements of serum creatinine at baseline and 2 years after the enrollment, extracting 701 patients (Fig 1). The demographic characteristics included sex, age, body mass index (BMI), original kidney disease (diabetic nephropathy, hypertensive nephropathy, glomerulonephritis, polycystic kidney disease, solitary kidney and others), systolic blood pressure (SBP) and baseline estimated GFR. Blood parameters included hemoglobin (Hb), white blood cell (WBC), platelet (Plt), albumin (Alb), uric acid (UA), sodium (Na), potassium (K), chloride (Cl), Na-Cl (as a surrogate of HCO_3_^-^), albumin-corrected calcium (cCa), inorganic phosphorus (P), low-density lipoprotein cholesterol (LDL-C) and C-reactive protein (CRP). Urine parameters included spot urine proteinuria (expressed as gram per gram creatinine excretion) and spot urine hematuria by dipstick (coded as four grades of 0 to 3 according to 0, 1+, 2+, and 3+ and as 0.5 if ±). Blood was tested using hematology autoanalyzer (Sysmex XE-5000, Kobe, Japan) and blood chemistry parameters were measured by routine measurements using autoanalyzer (LABOSPECT 008, Hitachi High-Technologies Corporation, Tokyo, Japan). Of note is that creatinine concentration in serum and urine was measured by an enzymatic method and urinary protein concentration measured by a pyrocatechol violet-metal complex assay method.

The time-varying parameters except for estimated GFR were calculated as time-averaged values over 2 years using a trapezoidal rule, giving rise to a 2-year time-averaged dataset. Percentage change in estimated GFR was calculated as: (estimated GFR at 2 year − baseline estimated GFR) / (baseline estimated GFR) × 100% [18].

Drug use of antihypertensives including angiotensin converting enzyme inhibitor or angiotensin II receptor blocker (combined as RASi), calcium channel blocker (CCB), diuretics or others were recorded as yes (coded as 1) or no (coded as 0). Estimated GFR was calculated using the Modification of Diet in Renal Disease (MDRD) study equation for Japanese population [19]. And the grade of chronic kidney disease (CKD) was classified based on the Kidney Disease Outcomes Quality Initiative (K/DOQI) practice guidelines [12].

### End points of renal outcomes

A primary end point was defined as reaching ESRD (initiation of hemodialysis or peritoneal dialysis). Death was treated as censoring because the present study solely focused on the risk factors of CKD progression rather than the risk of mortality. In addition, the degree of % decline in estimated GFR over 2 years was analyzed if it could efficiently predict the subsequent ESRD. In particular, 30% decline in estimated GFR over 2 years was set as a novel end point according to the report by Coresh [18].

### Statistical analyses

All continuous data are presented as mean ± standard deviation (SD). Differences in continuous variables between baseline dataset and 2-year dataset were compared by paired t test or Wilcoxon signed rank test depending on the distribution of the data. Time-to event analysis was performed using Cox proportional hazard model. Parameters were first tested for their proportional hazards assumption using both a time-dependent Cox regression and Schoenfeld residual plot. If time-dependency existed, a multivariate Cox regression analysis was performed by adjusting for an interaction term with a Heaviside function of time. A multivariate Cox regression was done in a stepwise manner with inclusion of *p* < 0.05 and exclusion of *p* > 0.10. Goodness-of-fit of the proposed model was measured by Akaike information criterion (AIC) [20,21].

To assess the usefulness of 30% decline in estimated GFR over 2 years as a novel surrogate predictor, we used a multivariate Cox regression model therewith. The association with 30% decline in estimated GFR over 2 years was examined by a multivariate logistic regression analysis. The significant predictor for this novel end point was subjected to receiver operating characteristic (ROC), showing the area under curve (AUC) with its 95% confidence interval (95% CI) and the cut-off point of the predictor [22,23]. All statistical analyses were performed using SPSS version 22 (IBM, Tokyo). A *p* value less than 0.05 was considered statistically significant.

## Results

### Baseline characteristics and time-averaged values in the follow-up of 2 years

During the follow-up period (4.5 ±1.3 years), 83 out of 701 patients progressed to ESRD. The baseline characteristics in addition to time-averaged values over 2 years are shown in Table 1. The time-varying values in two datasets were compared by paired t test whereas changes in C-reactive protein, proteinuria and hematuria were tested by Wilcoxon signed rank test because the data did not obey the normal distribution. Statistical significances were obtained in many time-dependent covariates except for serum sodium, potassium and C-reactive protein (Table 1). It was characteristic that systolic blood pressure, hemoglobin, serum Na-Cl, albumin-corrected Ca and LDL-cholesterol decreased whereas uric acid, phosphorus and proteinuria increased over 2 years of the follow-up. Drug use of antihypertensive agents including RASi increased with time probably due to targeting optimal blood pressure.

**Table 1:** Baseline characteristics and the time-averaged values over 2 years of the CKD cohort (n = 701).

Characteristics	Baseline	Time-averaged over 2 years	*p* value^¶^
Age (y)	62.3±13.0	-	
Sex			
Male (%)	440 (62.8%)	-	
Female (%)	261 (37.2%)	-	
Baseline eGFR (mL/min/1.73m^2^)	41.8±12.8	-	
CKD stage			
3a (%)	329 (46.9%)	-	
3b (%)	219 (31.2%)	-	
4 (%)	153 (21.8%)	-	
Original kidney disease			
DMN (%)	159 (22.7%)	-	
HTN (%)	314 (44.8%)	-	
CGN (%)	157 (22.4%)	-	
PKD (%)	15 (2.1%)	-	
Others (%)	56 (8.0%)	-	
BMI (kg/m^2^)	24.4±4.4	24.4±4.5	0.511
SBP (mmHg)	136.9±20.0	134.2±14.5	< 0.001
Blood Parameters			
Hb (g/dL)	12.9±1.9	12.8±1.8	< 0.001
WBC (×10^2^)	64.6±20.4	63.6±17.2	0.020
Plt (×10^4^)	21.9±6.7	21.5±6.1	0.001
Alb (g/dL)	3.98±0.44	4.02±0.39	< 0.001
UA (mg/dL)	6.5±1.4	6.6±1.3	0.029
Na (mEq/L)	140.8±2.7	140.7±2.4	0.684
K (mEq/L)	4.47±0.51	4.48±0.43	0.520
Na-Cl (mEq/L)	35.5±2.4	35.0±2.4	< 0.001
cCa (mg/dL)	8.85±0.47	8.77±0.49	< 0.001
P (mg/dL)	3.36±0.51	3.43±0.42	< 0.001
CRP (mg/dL)	0.25±0.57	0.26±0.48	0.513
LDL-C (mg/dL)	110.6±30.3	106.4±25.3	< 0.001
Urine Parameters (spot)			
TPU/CrU (g/g Cr)	0.90±1.31	0.96±1.33	0.049
UB_score	0.51±0.82	0.42±0.62	< 0.001
Drug use			
RASi Y/N (%Y)	391 (55.8%)	472 (67.3%)	< 0.001
CCB Y/N (%Y)	270 (38.5%)	356 (50.8%)	< 0.001
Diuretic Y/N (%Y)	109 (15.5%)	152 (21.7%)	< 0.001
Other AHD Y/N (%Y)	44 (6.3%)	75 (10.7%)	< 0.001

The difference of means between two groups was analyzed by paired t test if the data showed normal distribution; otherwise, Wilcoxon singed rank test was employed for CRP, TPU/CrU, UB_score and Drug use.

^¶^ Paired t test or Wilcoxon signed rank test.

eGFR, estimated glomerular filtration rate; DMN, diabetic nephropathy; HTN, hypertensive nephropathy; CGN, chronic glomerulonephritis; PKD, polycystic kidney disease; BMI, Body Mass Index; SBP, systolic blood pressure; Hb, hemoglobin; WBC, white blood cell; Plt, platelet; Alb, albumin; UA, uric acid; Na, sodium; K, potassium; Cl, chloride; cCa, albumin-corrected calcium; P, phosphorus; CRP, C reactive protein; LDL-C, low-density lipoprotein cholesterol; UB_score, urine blood score; TPU/CrU, urine total protein divided by urine creatinine; RASi, RAS inhibitor; CCB, calcium channel blocker; other AHD, other antihypertensive drugs.

### Cox proportional hazard model using the baseline dataset

We performed the time-to-event analysis for reaching ESRD using 21 covariates including sex, age, BMI, diabetic kidney, baseline estimated GFR, SBP, Hb, WBC, Plt, Alb, UA, Na, K, Na-Cl, cCa, P, LDL-C, CRP, proteinuria, hematuria and use of RASi. First, the baseline parameters were tested one by one for the proportional hazard assumption using both a time-dependent Cox regression and Schoenfeld residual plot. The violation was not observed (data not shown). Then, a univariate Cox regression was performed for each covariate, disclosing that sex, age, diabetic kidney, baseline estimated GFR, SBP, Hb, Alb, UA, Na, K, Na-Cl, P, LDL-C, proteinuria, hematuria and use of RASi were *p* < 0.1 for statistical significance. Finally, a multivariate Cox regression revealed that baseline estimated GFR, proteinuria, albumin, diabetic nephropathy, LDL-C, age, Na-Cl and use of RASi were statistically significant in the descending order of Wald statistic (Table 2, Model 1).

**Table 2:** Cox regression for ESRD using baseline dataset (n = 701).

Model	AIC	Characteristic	β	Wald	Exp(β)	95% CI	*p* value
Model 1	828.9	Baseline eGFR	-0.10	64.27	0.91	0.88 to 0.93	< 0.001
		TPU/CrU_0	0.33	43.39	1.40	1.26 to 1.54	< 0.001
		Alb_0	-1.18	18.74	0.31	0.18 to 0.52	< 0.001
		DMN	0.68	8.72	1.97	1.26 to 3.08	0.003
		LDL-C_0	-0.01	6.52	0.99	0.98 to 1.00	0.011
		Age	-0.02	6.30	0.98	0.96 to 1.00	0.012
		Na-Cl_0	-0.12	5.58	0.89	0.81 to 0.98	0.018
		RASi_0	-0.50	4.75	0.61	0.39 to 0.95	0.029

“0” following the parameter denotes the baseline value.

A univariate Cox regression was performed and the parameter showing *p* < 0.1 underwent a multivariate Cox regression analysis in a stepwise manner.

Model 1: Baseline dataset (Sex, Age, DMN, Baseline eGFR, SBP_0, Hb_0, Alb_0, UA_0, Na_0, K_0, Na-Cl_0, P_0, LDL-C_0, TPU/CrU_0, UB_score_0, RASi_0).

### Percentage changes in estimated GFR over 2 years

**Fig 2:**
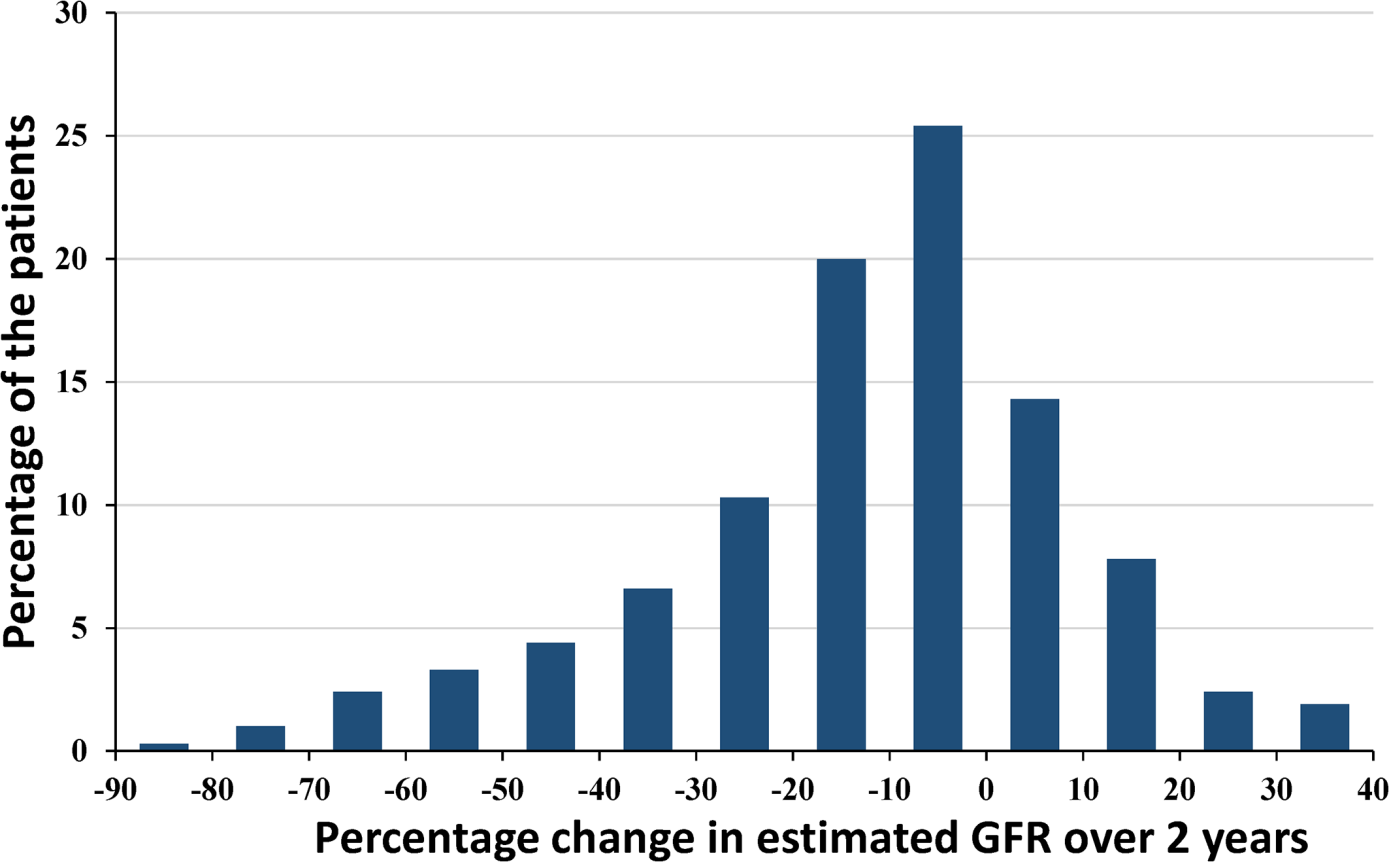
Distribution of percentage change in estimated GFR over 2 years. Percentage changes in estimated GFR were calculated with two measurements of serum creatinine at entry and after 2 years. The data show near normal distribution. The patients showing “rapid progression” defined as 50% decline over 2 years are observed in 7% of the total patients.

**Fig 3:**
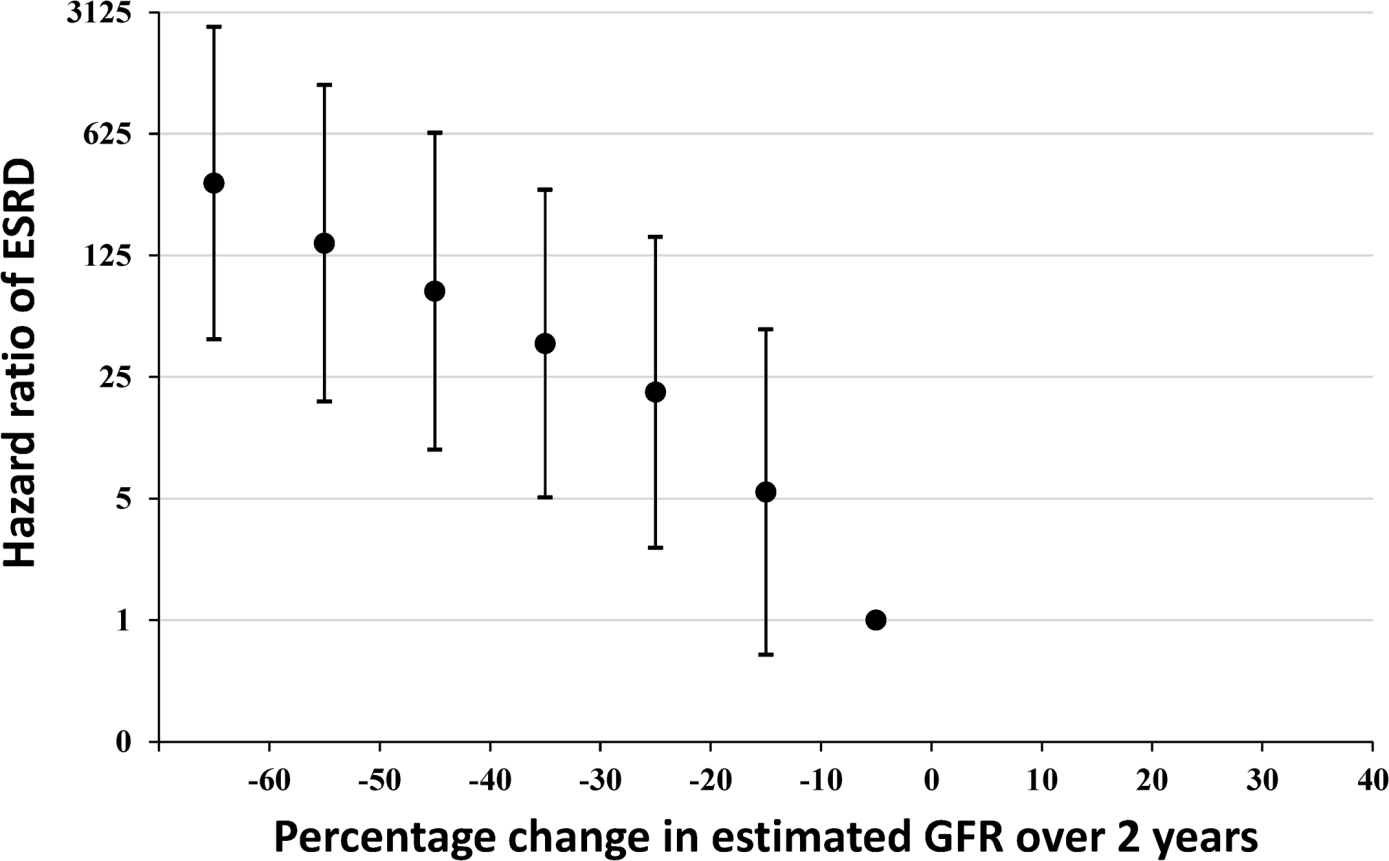
Hazard ratios of ESRD with every 10% decline in estimated GFR over 2 years. Hazard ratio reaching ESRD was determined by a Cox regression analysis by adjusting for age, sex, diabetic nephropathy and baseline estimated GFR as the baseline characteristics and 2-year time-averaged systolic blood pressure and proteinuria (total six confounders). Hazard ratios for subsequent risk of ESRD increase with every 10% decline in estimated GFR over 2 years in an exponential manner.

The principal aim of the present study was to test usefulness of 30% decline in estimated GFR over 2 years in predicting subsequent ESRD in our CKD cohort. Total patients were divided by every 10% change in estimated GFR as shown in Fig 2. Using 0 to 10% decline in estimated GFR over 2 years as a reference, hazard ratio reaching ESRD was determined by a Cox regression analysis in Fig 3. The result was adjusted for age, sex, baseline estimated GFR, diabetic nephropathy as demographic characteristics and for 2-year time-averaged systolic blood pressure and proteinuria (total six confounders). Hazard ratio for subsequent risk of ESRD increased with every 10% decline in estimated GFR over 2 years in an exponential manner (Fig 3). Hazard ratio of 30–40% decline in estimated GFR over 2 years showed 39 times greater than the reference (adjusted HR 38.9, 95%CI 5.1–298.1, *p* < 0.001). The incidence of ESRD (n = 17) was highest in this group in our cohort. Our results corroborated well with the recent study by Coresh et al. who reported that 30% decline in estimated GFR over 2 years showed the highest percentage of population attributable risk [18].

### Cox proportional hazard model using the 2-year time-averaged dataset

30% decline in estimated GFR over 2 years showed a crude hazard ratio of 31.60 when ESRD was an end point (Table 3, Model 1). Next, parameters of the 2-year time-averaged dataset were tested for proportional hazard assumption. The result indicated that phosphorus showed time-dependency; the interaction term of phosphorus x time showed β coefficient = -0.519 and *p* = 0.012. Schoenfeld residual plot showed negative correlation thus a Heaviside function was introduced to take on the value 1 if time < 3 years. Then, an extended Cox regression was performed, revealing that proteinuria, baseline estimated GFR, albumin, male, phosphorus and hemoglobin were significant (Table 3, Model 2). Time-dependency of phosphorus disappeared probably due to adjustment for other covariates. Finally, 30% decline in eGFR over 2 years was added, yielding the lowest AIC of 642.4 among these 3 models.

**Table 3:** Cox regression for ESRD using 2-year time-averaged dataset (n = 701).

Model	AIC	Characteristic	β	Wald	Exp(β)	95% CI	*p* Value
Model 1	809.7	30% decline in eGFR over 2 years	3.45	158.51	31.60	18.46 to 54.10	< 0.001
Model 2	769.8	TPU/CrU_2y	0.53	75.12	1.69	1.50 to 1.91	< 0.001
		Baseline eGFR	-0.09	42.55	0.92	0.89 to 0.94	< 0.001
		Alb_2y	-1.31	15.48	0.27	0.14 to 0.52	< 0.001
		Sex (Male)	0.96	13.66	2.60	1.57 to 4.32	< 0.001
		P_2y	0.99	11.87	2.70	1.54 to 4.76	0.001
		Hb_2y	-0.27	9.41	0.76	0.64 to 0.91	0.002
Model 3	642.4	30% decline in eGFR over 2 years	2.37	49.55	10.66	5.51 to 20.59	< 0.001
		Baseline eGFR	-0.07	29.44	0.93	0.91 to 0.96	< 0.001
		TPU/CrU_2y	0.28	11.44	1.33	1.13 to 1.57	0.001
		Alb_2y	-1.12	10.53	0.33	0.17 to 0.64	0.001
		Hb_2y	-0.20	5.49	0.82	0.69 to 0.97	0.019

“2y” following the parameter denotes the time-averaged value of 2 years.

A univariate Cox regression was performed and the parameter showing *p* < 0.1 underwent a multivariate Cox regression analysis in a stepwise manner.

Model 1: 30% decline in eGFR over 2 years alone.

Model 2: 2-year time-averaged dataset (Sex, Age, DMN, Baseline eGFR, SBP_2y, Hb_2y, Alb_2y, UA_2y, Na_2y, K_2y, Na-Cl_2y, cCa_2y, P_2y, CRP_2y, LDL-C_2y, TPU/CrU_2y, UB_score_2y).

Model 3: Model 1 + Model 2.

When compared between the baseline dataset and the 2-year time-averaged dataset, baseline estimated GFR and proteinuria were consistently chosen as progression factors to ESRD, and albumin was consistently selected as a protection factor to halt CKD from deteriorating. The impact of time-averaged phosphorus was stronger than previously thought because a 1 mg/dL increase of serum phosphorus in the follow-up predicted 2.7 times greater chance to reach ESRD (Table 3, Model 2).

**Fig 4:**
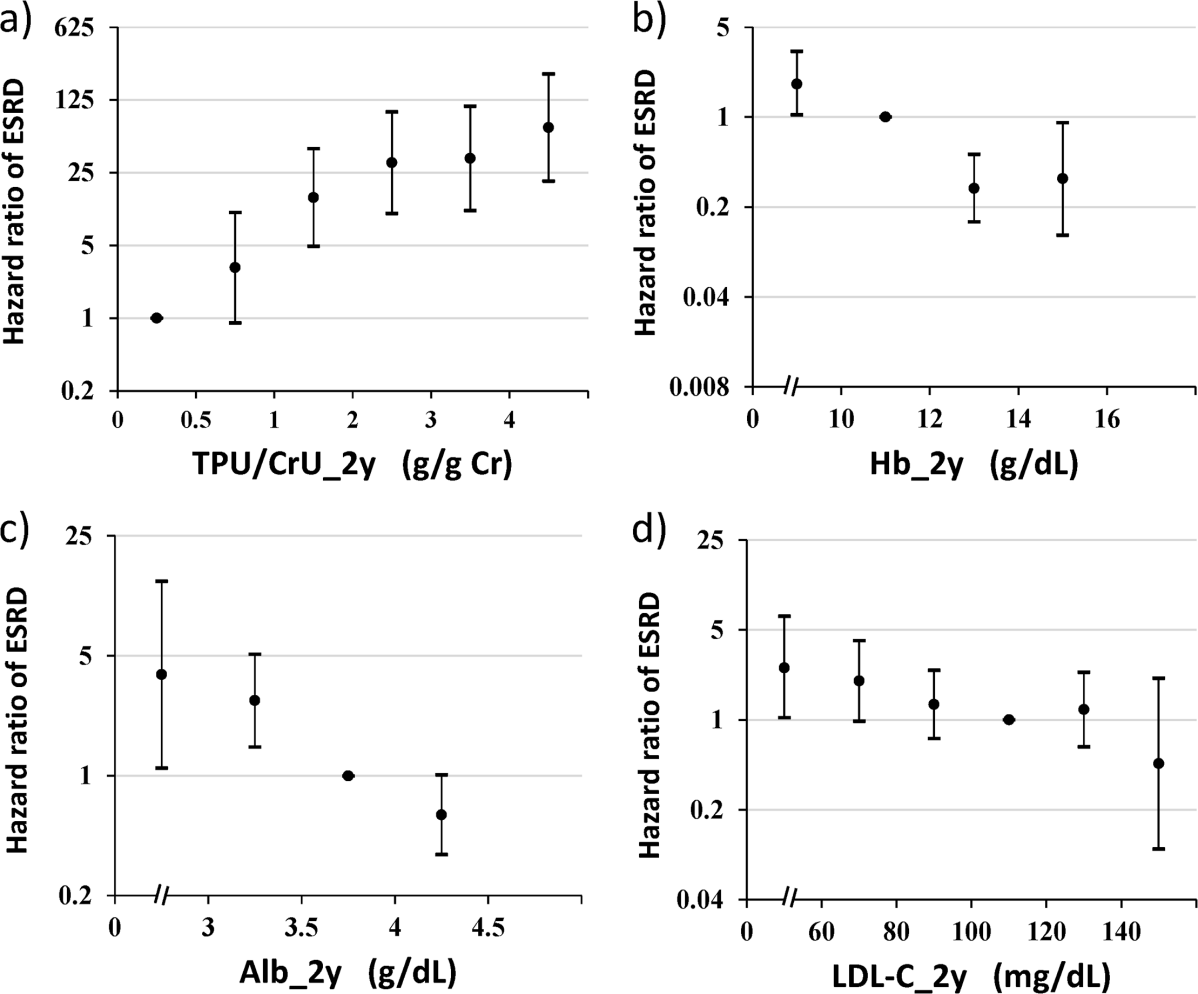
Hazard ratio of subsequent ESRD by time-averaged values over 2 years. Hazard ratios of several predictors for subsequent risk of ESRD are depicted by sectioning the time-averaged values over 2 years with the respective normal range as references. Hazard ratios are adjusted for six basic covariates such as sex, age, diabetic nephropathy, baseline estimated GFR and time-averaged systolic blood pressure and proteinuria over 2 years. **a**) Proteinuria with reference of < 0.5 g/g creatinine (adjustment for time-averaged proteinuria over 2 years was not done), **b)** Hemoglobin with reference of 10–12 g/dL, **c)** Serum albumin with reference of 3.5–4.0 g/dL, **d**) LDL-cholesterol with reference of 100–120 mg/dL.

The hazard ratio of several risk factors was depicted after sectioning the values with a reference of the normal range (Fig 4). Among the time-averaged values over 2 years the effects of proteinuria, serum albumin, hemoglobin and LDL cholesterol were selected. Proteinuria > 1 g/ g creatinine showed statistical significance in an exponential manner (Fig 4A). With respect to the effect of hemoglobin the result showed that hemoglobin < 10 g/dL was worse while hemoglobin >12 g/dL was better for the CKD progression with a reference of hemoglobin 10–12 g/dL (Fig 4B). Serum albumin showed that albumin < 3 g/dL was worse while albumin > 4.0 g/dL was better with a reference of albumin 3.5–4.0 g/dL (Fig 4C). LDL cholesterol was examined because baseline LDL cholesterol revealed a risk factor for ESRD (Table 2). The lower time-averaged LDL cholesterol < 60 mg/dL showed the higher hazard ratio as compared with the reference of 100–120 mg/dL (Fig 4D). The higher LDL cholesterol > 120 mg/dL did not show the significant effect.

### Logistic regression analysis for 30% decline in estimated GFR over 2 years

The impact of 30% decline in estimated GFR over 2 years prompted us to explore the association study. By inclusion criteria censoring did not occur during 2 years. Using the baseline dataset having 21 covariates for adjustment, many parameters such as baseline estimated GFR, proteinuria, serum albumin, diabetic nephropathy, phosphorus, age, use of RASi, systolic blood pressure and male were significantly associated with 30% decline in estimated GFR over 2 years (n = 126) (Table 4, Model 1). Using the 2-year time-averaged dataset, proteinuria, hemoglobin, uric acid, phosphorus, male, age systolic blood pressure and use of RASi were chosen as significant covariates (Table 4, Model 2). AIC dramatically went down from 468.4 to 395.9, suggesting that the model using the 2-year time-averaged dataset fitted better. The result was confirmed by Nagelkerke R^2^ (Pseudo R^2^ increased from 42% to 54%). Of interest is that in the Model 2 baseline estimated GFR disappeared and uric acid appeared (Table 4, Model 2). Taken all together, it is safe to say that controlling proteinuria, anemia, hyperuricemia, hyperphosphatemia in addition to add-on therapy of RAS inhibitor can inhibit progressing to ESRD. However, how strictly targeting these risk factors is the next question.

**Table 4:** Logistic regression for 30% decline in estimated GFR over 2 years by baseline dataset and 2-year time-averaged dataset (n = 701).

Model	Nagelkerke R^2^	AIC	Characteristic	β	Wald	OR	95% CI	*p* value
Model 1	0.42	468.4	Baseline eGFR	-0.06	32.79	0.94	0.93 to 0.96	< 0.005
			TPU/CrU_0	0.52	30.85	1.68	1.40 to 2.01	< 0.001
			Alb_0	-1.05	13.81	0.35	0.20 to 0.61	< 0.001
			DMN	0.86	10.82	2.36	1.42 to 3.94	0.001
			P_0	0.79	9.93	2.20	1.35 to 3.60	0.002
			Age	-0.03	9.90	0.97	0.95 to 0.99	0.002
			RASi_0	-0.55	4.81	0.58	0.36 to 0.94	0.028
			SBP_0	0.01	3.94	1.01	1.00 to 1.02	0.047
			Sex(Male)	0.53	3.89	1.70	1.00 to 2.89	0.049
Model 2	0.54	395.9	TPU/CrU_2y	0.74	55.00	2.10	1.73 to 2.56	< 0.001
			Hb_2y	-0.48	29.48	0.62	0.52 to 0.73	< 0.001
			UA_2y	0.47	15.30	1.61	1.27 to 2.04	< 0.001
			P_2y	1.01	7.71	2.76	1.35 to 5.64	0.006
			Sex (Male)	0.81	6.23	2.24	1.19 to 4.21	0.013
			Age	-0.03	5.68	0.98	0.96 to 1.00	0.017
			SBP_2y	0.02	5.17	1.02	1.00 to 1.04	0.023
			RASi_2y	-0.57	4.40	0.56	0.33 to 0.96	0.036

“0” following the parameter denotes the baseline value; “2y” following the parameter denotes the time-averaged value over 2 years.

A univariate logistic regression was performed and the parameter showing *p* < 0.1 underwent a multivariate logistic regression analysis in a stepwise manner. OR: odds ratio.

Model 1: Baseline dataset (Sex, Age, DMN, Baseline eGFR, SBP_0, Hb_0, Alb_0, UA_0, Na_0, K_0, Na-Cl_0, P_0, LDL-C_0, TPU/CrU_0, UB_score_0, RASi_0).

Model 2: 2-year time-averaged dataset (Sex, Age, DMN, Baseline eGFR, SBP_2y, Hb_2y, Alb_2y, UA_2y, Na_2y, K_2y, Na-Cl_2y, P_2y, LDL-C_2y, TPU/CrU_2y, UB_score_2y, RASi_2y).

### ROC analysis of the 30% decline in estimated GFR

ROC curve analysis was employed to investigate the cut-off point of the predictors for 30% decline in estimated GFR over 2 years. For this purpose time-averaged values over 2 years were investigated and the results with AUC and cut-off point are shown in Table 5. Targeting these risk factors involves proteinuria < 1 g/g creatinine, hemoglobin > 12 g/dL, uric acid < 7 mg/dL, phosphorus < 3.5 mg/dL and systolic blood pressure < 135 mmHg (Table 5).

**Table 5:** Diagnostic performance of time-averaged values over 2 years for 30% decline in estimated GFR over 2 years (n = 701).

Characteristic	AUC	95% CI	Cut-off point
TPU/CrU_2y	0.84	0.79 to 0.88	1.05 g/g Cr
UA_2y	0.78	0.74 to 0.82	6.95 mg/dL
Hb_2y	0.75	0.70 to 0.80	12.3 g/dL
P_2y	0.72	0.67 to 0.77	3.51 mg/dL
Baseline eGFR	0.70	0.65 to 0.76	38.7 mL/min/1.73 m^2^
SBP_2y	0.66	0.61 to 0.71	135.1 mmHg

“2y” following the parameter denotes the time-averaged value of 2 years. The cut-off point of the parameter is shown with unit. Cr: creatinine.

## Discussion

The risk factors for ESRD were confirmed by time-to-event survival analysis by a Cox proportional hazard model using the baseline dataset and the 2-year time-averaged dataset. Those risk factors were consistently baseline estimated GFR, proteinuria, serum albumin irrespective of the dataset used. In the present discussion, we will focus on several risk factors commonly unveiled.

First of all, proteinuria was always one of the worst predictor, and the effect of proteinuria on the subsequent incidence of ESRD was dose-dependent with > 1 g/g creatinine. The mechanisms by which proteinuria aggravates kidney injuries are likely two folds. One possible explanation lies on the albumin-induced tubulo-interstitial injuries via cytokine-mediated mechanisms including the profibrogenic cytokine transforming growth factor β, monocyte chemoattractant-1, regulated upon activation, normal T cell expressed and secreted (RANTES), interleukin-8 fractalkine, and other mediators [24]. Another possibility may involve albumin-bound lipids, apoptosis, complement activation and autophagy due to filtered macromolecules besides albumin [25]. Targeting proteinuria should be achieved at 1 g/g creatinine or below.

Anemia is also one of the biggest risk factors for predicting ESRD. The mechanism of anemia to advance CKD attracted scientific attention and was attributed to interstitial fibrosis induced by hypoxia inducible factor and other mediators [26,27]. Many large-scaled clinical trials, however, did not reach consensus on the target range of the hemoglobin concentration [28,29] Recent meta-analysis of 19 trials with more than 8,000 participants failed to show the beneficial effects of higher hemoglobin on the renal outcome [30]. According to the present study, the adjusted hazard ratio of hemoglobin < 10 g/dL over 2 years significantly increased hazard ratio with a reference to 10–12 g/dL hemoglobin. On the other hand, the CKD patients having hemoglobin greater than 12–14 g/dL significantly decreased hazard ratio, suggesting some beneficial effect on inhibiting the start of ESRD. But the optimal range of hemoglobin should be determined in view of the adverse effects such as hypertension and thrombosis, and of health economics as well. Whether a higher level of hemoglobin (11–13 g/dL) protects the progression of non-diabetic CKD patients is under way by the study name of PREDICT (UMIN000006616).

Serum albumin was consistently chosen as a protective factor in the present study. The serum albumin decreases with the proteinuria as a mirror image but it may serve a renoprotective role independent of proteinuria because the significance was always adjusted for proteinuria. Hypoproteinemia has recently emerged as an important independent risk factor for kidney disease progression [20]. This notion has been emphasized by Ni et al [6] who recently reported that time-averaged albumin has an inhibitory impact on CKD progression similar to our result. One possible explanation is that albumin may bind to so-called uremic toxins, protecting the kidney from progressing to ESRD [31]. Low level of serum albumin also reflects the state of malnutrition and inflammation [32], thus the implication of hypoalbuminemia may serve as a multifactorial risk factor in CKD progression.

LDL cholesterol at the baseline was detected as an independent factor adjusted for serum albumin, proteinuria and hemoglobin in the present study. But the meaning is protective because β coefficient was minus, indicating of the protective role in CKD progression. The hazard ratio of the subsequent ESRD was significant in less than 60 mg/dL of LDL cholesterol over 2 years. The finding seems paradoxical but may be attributable to the so-called “reverse epidemiology” [10]. It is explained that lower level of LDL cholesterol is caused by confounders such as malnutrition and chronic inflammation. But the association between LDL cholesterol and C-reactive protein was not significant in the present study (data not shown). Another possibility is that higher level of LDL cholesterol was associated with higher chance of using statin which may work as renoprotection beyond the cholesterol-lowering effect [33]. Unfortunately, the information on using lipid-lowering agents including statin was not recorded. Further study is necessary to explore the enigma between CKD progression and LDL cholesterol.

The striking finding of the present study shows that 30% decline in estimated GFR over 2 years was quite useful for predicting the subsequent risk of ESRD. A crude hazard ratio of this novel surrogate predicted more than 30 times possibility to enter ESRD in the future. The finding was confirmed by a multivariate Cox regression analysis adjusted for baseline estimated GFR, proteinuria, albumin and hemoglobin (these are classical major risk factors). Our result validated the utility of this novel predictor recently advocated by Coresh and colleagues [18]. We extended the association analysis of the parameters with 30% decline in estimated GFR over 2 years by a multivariate logistic regression analysis because censoring did not occur during 2 years. Proteinuria, phosphorus, hemoglobin and uric acid were found independent parameters. The results suggest that intervening these risk factors may inhibit reaching this end point, finally enabling to inhibit the progression to ESRD. Although this novel surrogate appears superior to other possible predictors, a disadvantage seems to wait for 2 years to calculate the parameter.

Nonetheless, we have to discuss about several limitations in the present study. First of all, the number of the patients are relatively small especially when dividing to several subgroups. Second, the history of drug use should be recorded in addition to antihypertensive drugs. The information of use of statin, antiplatelet agents and the medication for CKD-MBD may further clarify the pathophysiology of CKD progression. Although with these limitation, the present study may have some strengths because all the time-varying covariates were estimated as time-averaged values over 2 years, better representing the continued exposure status to the risk factors [6,34–37]. The present study could show a better model of risk factors for CKD progression using a 2-year time-averaged dataset than using a baseline dataset. These findings facilitate the interventional treatments for many modifiable risk factors in clinical practice. Of more importance is that usefulness of 30% decline in estimated GFR over 2 years was verified by the survival analysis. Using this novel surrogate, clinicians are able to predict the subsequent occurrence of ESRD more efficiently than ever, helping practice evidence-based medicine.

## Conclusion

Survival analysis showed that baseline estimated GFR, serum albumin and proteinuria are the three major predictors of CKD not only at baseline but also in 2-year follow-up. Hemoglobin becomes a predictor in the follow-up. Proteinuria less than 1 g/g creatinine and hemoglobin greater than 12 g/dL should be achieved to stabilize the clinical course of CKD. 30% decline in estimated GFR over 2 years is a promising novel surrogate for a future risk of ESRD.
